# Modifications in Rat Plasma Proteome after Remote Ischemic Preconditioning (RIPC) Stimulus: Identification by a SELDI-TOF-MS Approach

**DOI:** 10.1371/journal.pone.0085669

**Published:** 2014-01-13

**Authors:** Pierre Hibert, Delphine Prunier-Mirebeau, Olivia Beseme, Maggy Chwastyniak, Sophie Tamareille, Florence Pinet, Fabrice Prunier

**Affiliations:** 1 L’UNAM Université, Angers, France; 2 Université d’Angers, Laboratoire Cardioprotection Remodelage et Thrombose, Angers, France; 3 Université d’Angers, INSERM U771, CNRS UMR 6214, CHU Angers, Département de Biochimie et Génétique, Angers, France; 4 INSERM, U744, Lille, France; 5 Institut Pasteur de Lille, Lille, France; 6 Université Lille Nord de France, IFR142, Lille, France; 7 Centre Hospitalier régional et Universitaire de Lille, Lille, France; 8 CHU Angers, Service de Cardiologie, Angers, France; I2MC INSERM UMR U1048, France

## Abstract

Remote ischemic preconditioning’s (RIPC) ability to render the myocardium resistant to subsequent prolonged ischemia is now clearly established in different species, including humans. Strong evidence suggests that circulating humoral mediators play a key role in signal transduction, but their identities still need to be established. Our study sought to identify potential circulating RIPC mediators using a proteomic approach. Rats were exposed to 10-min limb ischemia followed by 5- (RIPC 5′) or 10-min (RIPC 10′) reperfusion prior to blood sampling. The control group only underwent blood sampling. Plasma samples were isolated for proteomic analysis using surface-enhanced laser desorption and ionization - time of flight - mass spectrometry (SELDI-TOF-MS). A total of seven proteins, including haptoglobin and transthyretin, were detected as up- or down-regulated in response to RIPC. These proteins had previously been identified as associated with organ protection, anti-inflammation, and various cellular and molecular responses to ischemia. In conclusion, this study indicates that RIPC results in significant modulations of plasma proteome.

## Introduction

First described in 1986 by Murry *et al*. [1], ischemic preconditioning (IPC) is a powerful technique, consisting of short periods of coronary artery ischemia-reperfusion (I/R), that attenuate I/R injuries caused by subsequent prolonged coronary occlusion. IPC can be effective in humans but requires invasive procedure, which could be harmful in clinical settings [2], [3]. Many experimental studies have therefore explored another preconditioning method, easily applicable to most patients: remote ischemic preconditioning (RIPC). This is a phenomenon whereby transient ischemia of a tissue or an organ at a distance from the heart affords cardioprotection when applied before myocardial I/R [4]. In this way, many remote tissues, such as the small intestine [5], kidney [6], or skeletal muscle [7], have shown potential to reduce subsequent myocardial infarct size. Furthermore, RIPC, which uses a standard blood pressure cuff placed around the arm, has emerged as an attractive non-invasive strategy in clinical settings [8], [9]. This approach has been taken in several clinical trials, and has been shown to be effective in patients undergoing corrective cardiac surgery for congenital heart disease [10], coronary bypass surgery [11], [12], elective surgery for abdominal aortic aneurysm [13], or elective percutaneous coronary intervention (PCI) [14]. More recently, RIPC was also demonstrated to significantly decrease myocardial injury when applied in the ambulance during transfer to primary PCI [15]. While the protective effects of RIPC are well-established, the underlying mechanisms involved remain unknown. Dickson *et al.* [16] were the first to demonstrate that a blood transfer from a remote preconditioned rabbit to a naive one exhibits protective benefits. This humoral cardioprotective phenomenon has since been observed in other animal models, and includes coronary effluent transfer [17] or RIPC cardioprotection of the recipient following brain-dead donor heart transplantation [18]. Shimizu *et al.* [19] have even shown that cross-species protection via RIPC is possible by protecting rabbit cardiomyocytes with human blood. Although these experiments are clear evidence that circulating humoral mediators play a major role in signal transduction [20], the identities of these effectors remain undetermined. In the past, many proteomic techniques were used to seek out novel biomarkers associated with cardiovascular diseases, such as left ventricular remodelling after a first myocardial infarction [21], [22] or abdominal aortic aneurysm [23]. In cardioprotection, a study using two-dimensional gel electrophoresis (2-DE), matrix assisted laser desorption and ionization – time of flight – mass spectrometry (MALDI-TOF-MS), and liquid chromatography – electrospray ionization – tandem mass spectrometry (nanoLC-ESI-MS/MS), was conducted to identify humoral factors involved in RIPC, but without success [24]. Surface-enhanced laser desorption and ionization - time of flight - mass spectrometry (SELDI-TOF-MS), is another proteomic method that combines both chromatography on ProteinChip arrays and mass spectrometry (MS), providing a proteomic high-throughput strategy for profiling potential humoral factors of RIPC [25]. Therefore, our research was designed to identify potential RIPC-induced humoral mediators using SELDI-TOF-MS technology.

## Materials and Methods

### Ethics Statement

All experiments were performed in accordance with the *Guide for the Care and Use of Laboratory Animals* published by the US National Institute of Health [NIH publication 85 (23), revised in 1996]. Protocols have been approved by our regional ethic committee: Comité Régional d’Ethique pour l’Expérimentation Animale-Pays de la Loire (CEEA.2012.50). All surgery was performed under sodium pentobarbital anesthesia, and all efforts were made to minimize suffering.

### Animal Studies

Wild type male Wistar rats, 8- to 10-week-old, were anesthetized with an intraperitoneal injection of sodium pentobarbital at a dose of 60 mg/kg (Ceva santé Animal, France). Animals were orotracheally intubated with a 16-gauge tube and ventilated using a small animal ventilator at a rate of 50–60 breaths per minute (SAR-830 A/P, CWE). Body core temperature was continuously monitored during the procedure and maintained at 37±1°C using a homeothermic blanket, connected to a temperature control unit (HB101/2 RS, Bioseb, France). RIPC was achieved by occlusion arterial blood flow for 10 minutes, using a vascular clamp placed on the upper right femoral artery, followed by reperfusion. Limb ischemia was confirmed by a change in skin color and a decrease in subcutaneous limb temperature. Following limb reperfusion, the skin color turned back to a pink color, while the under-skin temperature reached the baseline temperature. Rats were randomly assigned to one of three groups: RIPC 5′, with blood sampling 5 minutes after the end of limb ischemia (n = 10); RIPC 10, with blood sampling 10 minutes following the end of limb ischemia (n = 10); Control (no intervention), with blood sampling only (n = 10). Vena cava blood samples were collected at 5 or 10 minutes after limb reperfusion in glass tubes containing ethyldiamine tetraacetic acid (EDTA) as anticoagulant. Samples were centrifuged at 3000 rpm for 15 min and plasma was taken and immediately processed, divided into aliquots of 500 µL, and stored at −80°C. These samples underwent no more than two freeze/thaw cycles prior to analysis.

### Sample Treatment

Combinatorial peptide ligand library (CPLL) with ProteoMiner protein enrichment kit (Bio-Rad Laboratories, Hercules, CA) was used to pre-treat plasma samples as previously described [25]. These hexapeptides beads bind all the plasma proteins equally and allow to eliminate the excess of unbound majority proteins. The low-abundance plasma proteins are enriched compared to the high-abundance proteins which are in smaller quantities. The dynamic range is thus reduced. Columns containing these beads were previously washed and 200 µL of samples was then added and rotated end to end for 2 h at room temperature. Unbound proteins were then eliminated and beads were washed. Sample was then eluted by adding 20 µL elution reagent, repeated three times. Elution samples obtained from all 3 centrifugations were pooled and stored at −20°C.

### SELDI-TOF-MS Profiling

SELDI-TOF-MS analysis was used to profile samples collected for the selection of potential biomarkers of remote conditioning. Samples were profiled with eight spot format ProteinChip Arrays CM10 (Weak Cation Exchanger) or H50 (Reverse Phase) (Bio-Rad Laboratories). Arrays were prepared with 5 µL samples diluted 10-fold in binding buffer, which was sodium acetate 100 mmol/L pH4 for CM10 and ACN10%, TFA 0.1% and NaCl 150 mmol/L for H50 array. Native and CPLL treated forms of all samples were analyzed using the two types of ProteinChip and each of them was tested in duplicate and randomly distributed on arrays, as previously described [25]. All data were processed with the ProteinChip Data Manager software. For the peak selection of potential biomarkers, peaks were detected automatically: groups of peaks of similar mass across spectra were assembled into clusters, according to two-step parameter settings as previously described [25]. For the first step, peaks were automatically detected according to the specified S/N (4 or 5) and the minimum valley depth (3 or 4), if they were found in at least 10% of all spectra, with an *m/z* error of less than 0.2%. Settings for the second step were a S/N of 2 and a minimum valley depth of 2. The *m/z* range was set between 3000 and 30,000 for low-mass and between 20,000 and 150,000 for high-mass proteins. Clusters of all spectra were then submitted to a univariate analysis with a non-parametric test to calculate the p-value of each cluster. A Mann-Whitney test was used to compare groups in pairs and a Kruskal-Wallis to compare the three groups. Duplicates were averaged before any statistical analysis.

### Protein Sample Purification

Albumin and IgG were depleted, from samples not pre-treated by CPLL, with the ProteoPrep Blue Albumin Depletion Kit (Sigma-Aldrich) before protein separation by MicroRotofor cell or spin columns. After equilibration of the column, 25 µL of plasma was added; the sample was bound and then washed with equilibration buffer. The flow-through obtained from centrifugations of the depleted sample (125 µL) was then pooled and stored at −20°C.

In accordance with a previous study [26], we chose an adapted strategy for enrichment and purification of peaks selected by SELDI-TOF-MS, in order to identify them by MALDI-MS/MS. A set of techniques was used.

#### Liquid-phase isoelectric focusing

Proteins were isolated and purified by liquid-phase isoelectrofocusing (IEF) with the MicroRotofor cell (Bio-Rad Laboratories), according to their isolectric points. 1–2 mg of protein samples were diluted in IEF buffer (7 mol/L urea, 2 mol/L thiourea, CHAPS 4% [w/v] and 0.24% Triton X100), glycerol 5% [v/v] and ampholytes (appropriate pH gradient, 1.6% [v/v]). The MicroRotofor cell’s focusing chamber was loaded with 2.5 mL of samples and the electrode assemblies were filled with 6 mL of appropriate electrolytes (depending of the pH range investigated). Focusing was performed at room temperature, under a constant power of 1 W. At the end of the IEF, protein fractions from each compartment (200 µL) were harvested quickly to avoid the diffusion of separated proteins and stored at −20°C. Detection of peaks was performed on a ProteinChip NP20 array.

#### Protein treatment

Before SELDI-TOF-MS analysis of fractioned proteins, the 2-D Clean-Up kit (GE Healthcare) was used to precipitate proteins, and precipitation was performed to selectively discard contaminant from proteins. 300 µL of precipitant and co-precipitant were added to samples (1–100 µg protein), which were then centrifuged. The supernatant was removed and the protein pellet was washed by 40 µL of co-precipitant to remove more non-protein contaminants. After a second centrifugation, the pellet was finally suspended in 25 µL of deionized water, 1 mL of wash buffer and 5 µL of wash additive. Proteins from samples were too diluted for separation on spin columns. A precipitation was thus realized for 30 min at −20°C to concentrate proteins from samples. The mixture was then centrifuged and the pellet was finally dissolved in 20 µL of deionized water and stored at −20°C.

#### Gel electrophoresis

Fractions (or pooled fractions) containing the purified peak of interest were separated with SDS-PAGE gels with an appropriate percentage of acrylamide depending on the mass of the peak of interest (Bio-Rad Laboratories). Gels were then stained with Coomassie Brilliant Blue, as described by Neuhoff *et al.* [27].

### Protein Identification

#### Trypsic digestion

Proteins were then identified by an in-gel digestion method, as previously described [28]. Briefly, the band of interest was excised and the gel plugs were washed with ultrapure water until totally destained. Gel pieces were then rinsed several times with 50 mM ammonium bicarbonate and acetonitrile (ACN), before addition of 3 µL of 40 µg/mL trypsin (Trypsin Gold, Promega, Madison, WI) in 50 mmol/L acetic acid and 0.025% ProteasMAX™ Surfactant (Trypsine Enhancer, Promega, Madison, WI). After digestion overnight at 37°C, the supernatant was removed, and the gel pieces were washed with CAN [v/v]/0.1% TFA. Supernatants were desalted and eluted with ZipTip C18 in accordance with the manufacturer’s protocol (Millipore, Bedford, MA). 0.5 µL of this solution was then mixed with 0.5 µL of matrix solution (5 mg/mL of α-cyano-4-hydroxycinnamic acid dissolved in 10% TFA/50% ACN) and spotted onto the MALDI-TOF-MS target.

#### MALDI-TOF profiling

External calibration was performed with a peptide mixture resultant from the tryptic digest of BSA (0.5 µg/mL). MALDI-TOF-MS was then performed with a Voyager DE STR mass spectrometer (PerSeptive Biosystems, Framingham, MA, USA) equipped with a 337.1 nm nitrogen laser and a delayed extraction facility (125 msec). All spectra were acquired in a positive ion reflector mode at the voltage of 20 kV, with grid-voltage of 61%. Typically, 300 laser shots were recorded per sample. Following this, the mass spectra were calibrated prior to protein identification by peptide mass fingerprinting, conducted by running the MASCOT web searcher (http://www.matrixscience.com/, Matrix Science, UK) against the Swissprot 57-15 (515203 sequences; 181334896 residues) with the following parameters: fixed modifications: carbamidomethyl (C) and variable modifications: oxidation (M); peptide mass tolerance: ±50 ppm; peptide charge state: 1+; max missed cleavages: 1; taxonomy: rattus.

### Verification of Identification by Immunodepletion

After identification by MALDI-TOF-MS, validation was performed by immunodepletion using specific antibodies against the protein identified. 500 µL of RIPA buffer (Triton X-100 1%, NaCl 150 mmol/L, EDTA 1 mmol/L, EGTA 1 mmol/L, sodium vanadate 0.1 mmol/L, and NP40 0.5% in Tris-HCl 10 mmol/L) was added by 1 µL of native plasma sample and 5 or 10 µg of antibodies, as previously described [22]. The antibodies used were for haptoglobin (SantaCruz sc-134466), hemoglobin (SantaCruz sc-31116), apolipoprotein C-III (Tebu-bio, 036sc-50378), transthyretin (Abcam, ab905), apolipoprotein A-IV (SantaCruz sc-19036) and fibrinogen (sc-335581). After overnight incubation at 4°C, 50 µL of protein A Sepharose 4 Fast Flow beads (GE Healthcare) were added and the preparation was rotated end-to-end 4 h at 4°C. Supernatant was next obtained after centrifugation, and was precipitated in cold acetone overnight. Supernatant was removed after centrifugation 20 min at 12,000 g and pellet was dried 5 min at room temperature and added by 5 µL of SELDI binding buffer (depending on the array). Immunodepleted and undepleted plasma were compared by SELDI-TOF-MS on ProteinChip CM10 or H50 arrays and disappearance of the peak of interest was visualized, compare to untreated plasma.

### Statistical Analysis

All values were expressed as mean ±SEM. Clusters of all spectra obtained using SELDI-TOF-MS were subjected to univariate analysis, with a non-parametric test to calculate the *p* value of each cluster. A Mann-Whitney test was used to compare groups in pairs and a Kruskal-Wallis test to compare the three groups. A *p* value <0.05 was considered statistically significant.

## Results

### SELDI-TOF-MS Profiling of Plasma in RIPC-induced Circulating Factors in Rats

Plasmas from three groups of rats were analysed for differential proteomic analysis. The three groups are: Control (CRTL), which endured no intervention (except blood sampling), RIPC 5′ and RIPC 10′ groups, which endured blood sampling respectively 5 and 10 minutes after the end of limb ischemia.

All plasmas (n = 10 per group) were treated by CPLL as previously described [25]. The protein profiling of CPLL-treated and untreated plasma from the different groups was performed in duplicate with SELDI-TOF-MS technology on CM10 and H50 arrays.

**Figure 1:**
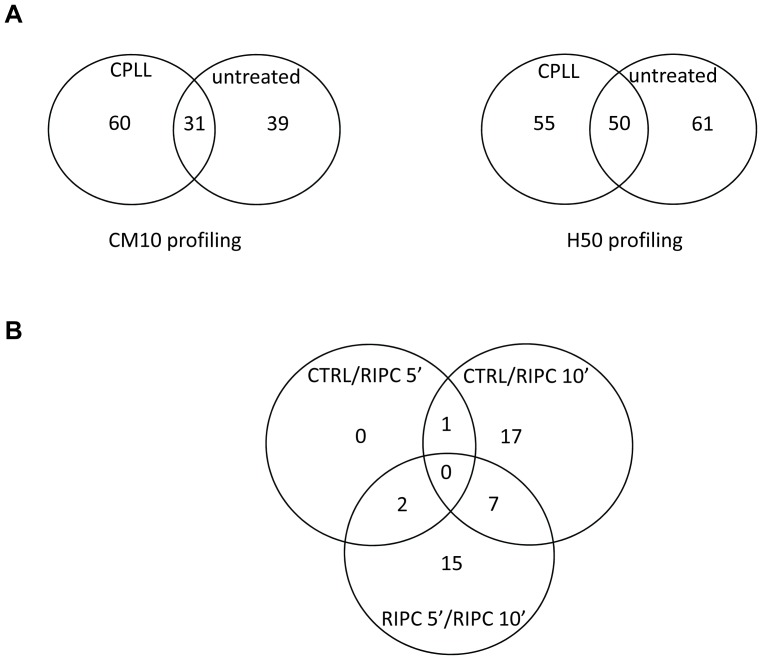
Venn diagram demonstrating the number of SELDI peaks detected and analysed for the comparison between the CRTL, RIPC 5′ and RIPC 10′ groups. **A:** Comparison of the number of peaks detected before or after CPLL treatment of plasma samples in CM10 and H50 arrays. **B:** Comparison of the number of peaks with significant modulations of intensity in the CRTL, RIPC 5′ and RIPC 10′. All the samples were analyzed in duplicate.

Using the clustering parameters described in Materials and methods section, we detected in the three groups of rats, a total of 130 peaks for CM10 arrays and 166 peaks for H50 arrays. Interestingly, we observed with the two arrays an increased number of peaks detected after CPLL treatment with 60 additional peaks in CM10 and 55 additional peaks in H50 arrays (**Figure 1A**) representing respectively 46% in CM10 arrays and 33% in H50 arrays of the total peaks detected.

SELDI-TOF analysis revealed 42 significant (p<0.05) modulations of peak intensity between two groups of rats with 18 down-regulated, 21 up-regulated and 3 down- or up-regulated (7192, 21120 and 140846 *m/z*) depending on the groups (**Table 1**). Only 3 peaks were found to be significantly expressed between CRTL and RIPC 5′ groups, 25 peaks between CRTL and RIPC 10′ groups and 24 peaks between RIPC 5′ and RIPC 10′ groups (**Figure 1B**). Interestingly, no peaks differentially expressed were in common between the three groups compared in pairs. In addition, only few peaks were differentially expressed between each pair of groups (**Figure 1B**).

**Table 1:** SELDI peaks selected to be differentially expressed.

CRTL/RIPC 5′				CRTL/RIPC 10′				RIPC 5′/RIPC 10′	
*Down-*	*Up-regulated*			Down-		Up-regulated		Down-	Up-regulated
			4386						
			6560						
					7017				
					7140				7140
7192									7192
					7227				
					8224				
					8317				
					8405				
					8527				
			9420						
					9566				9566
13038			13038						
			13156						
			13341						
							13720		
							13794		
							13902		
									15870
									15980
			21120						21120
			21182						
							21354		
			21390						
							26026		
					27340				
									27583
							27791		
			28284						
					29133				
					42427				42427
					42463				42463
									42610
									43655
									54700
					76060				76060
							87765		
									102847
			109310				109310		
							115169		
					138820				
140846									140846

Only peaks with statistical difference expression (p<0.05) between two groups are indicated and expressed in m/z.

Peaks outlined are common between two groups.

Peaks underlined are impossible to purify for protein identification.

**Table 2:** SELDI profiles of plasma from CRTL, RIPC 5′ and RIPC 10′ rats.

Protein name	Molecular mass (Da)	CTRL(n = 10)	RIPC 5′ (n = 10)	RIPC 10′ (n = 10)	Statistical analysis#	Peaks*m/z (Da)	Accession number
Apolipoprotein C-III	11117	4.98±2.41	3.43±1.40	2.89±0.79	CRTL *vs* RIPC 10′ (p = 0.02)	8317	P06759
Haptoglobin alpha chain	9317	8.53±1.28	9.38±1.68	9.50±0.66	CRTL *vs* RIPC 5′ *vs* RIPC 10′ (p = 0.056)	9420	P00738
Transthyretin (*monomeric form*)	15720	1.77±0.37	1.59±0.27	1.87±0.31	CRTL *vs* RIPC 10′ (p = 0.059)	13720	P02767
		0.55±0.31	0.64±0.35	0.40±0.17	RIPC 5′ *vs* RIPC 10′ (p = 0.059)	15870	
Hemoglobin beta chain	15979	0.87±0.52	0.93±0.46	0.62±0.27	RIPC 5′ *vs* RIPC 10′ (p = 0.059)	15980	P02091
Transthyretin(*dimeric form*)	31440	70.09±4.67	66.36±4.81	66.12±5.81	CRTL *vs* RIPC 10′ (p = 0.028)CRTL *vs* RIPC 5′ *vs* RIPC 10′ (p = 0.066)	27340	P02767
		1.61±0.24	1.69±0.29	1.41±0.21	RIPC 5′ *vs* RIPC 10′ (p = 0.049)	42427	
Apolipoprotein A-IV	44456	0.39±0.12	0.33±0.06	0.30±0.08	CRTL *vs* RIPC 10′ (p = 0.059)	42463	P02651
		1.02±0.14	1.06±0.17	0.89±0.13	RIPC 5′ *vs* RIPC 10′ (p = 0.059)	42610	P
Fibrinogen beta chain	50671	6.46±0.86	7.09±1.47	6.23±1.07	RIPC 5′ *vs* RIPC 10′ (p = 0.028)CRTL *vs* RIPC 5′ *vs* RIPC 10′ (p = 0.071)	54700	P14480

only the peaks with protein identification validated are listed.

^#^ Mann-whitney test was used to compare 2 groups and Kruskal-Wallis test for the 3 groups.

### Protein Identification of the Peaks Differentially Expressed between the Three Groups of Rats

We followed the same strategy for purification and mass spectrometry identification of SELDI peaks corresponding to low-abundance proteins that has been described previously [26]. The first step was the choice of the appropriate sample containing the selected peaks. From our databank recording all the *m/z* peaks detected from samples in different conditions, we excluded 12 peaks of our proteomic profiling to be purified (**Table 1**) due to low abundance in the sample or too much high intensity of peaks surrounded the peak to be purified. For the 6560, 7017, 7227, 8405, 8527, 9566, 13038, 13156, 13794, 21120, 21182, 21354, 21390 and 26026 *m/z* peaks, we used the strategy described previously [26] but we were unsuccessful to purify them and identify the corresponding protein.

Three SELDI peaks, 27583/27791/28284 have already been identified and validated to be apolipoprotein A-I [29].

For the other peaks (8317, 13720, 27340, 42427–42463, 42610 and 54700 *m/z*), the strategy already published was used and successful [26]. Detailed information on these peaks with significant mean intensity levels between the CRTL, RIPC 5′ and RIPC 10′ groups are provided on **Table 2**. Complete purification and protein identification process of the 8317 *m/*z peak is detailed below and is representative of the strategy used.

**Figure 2:**
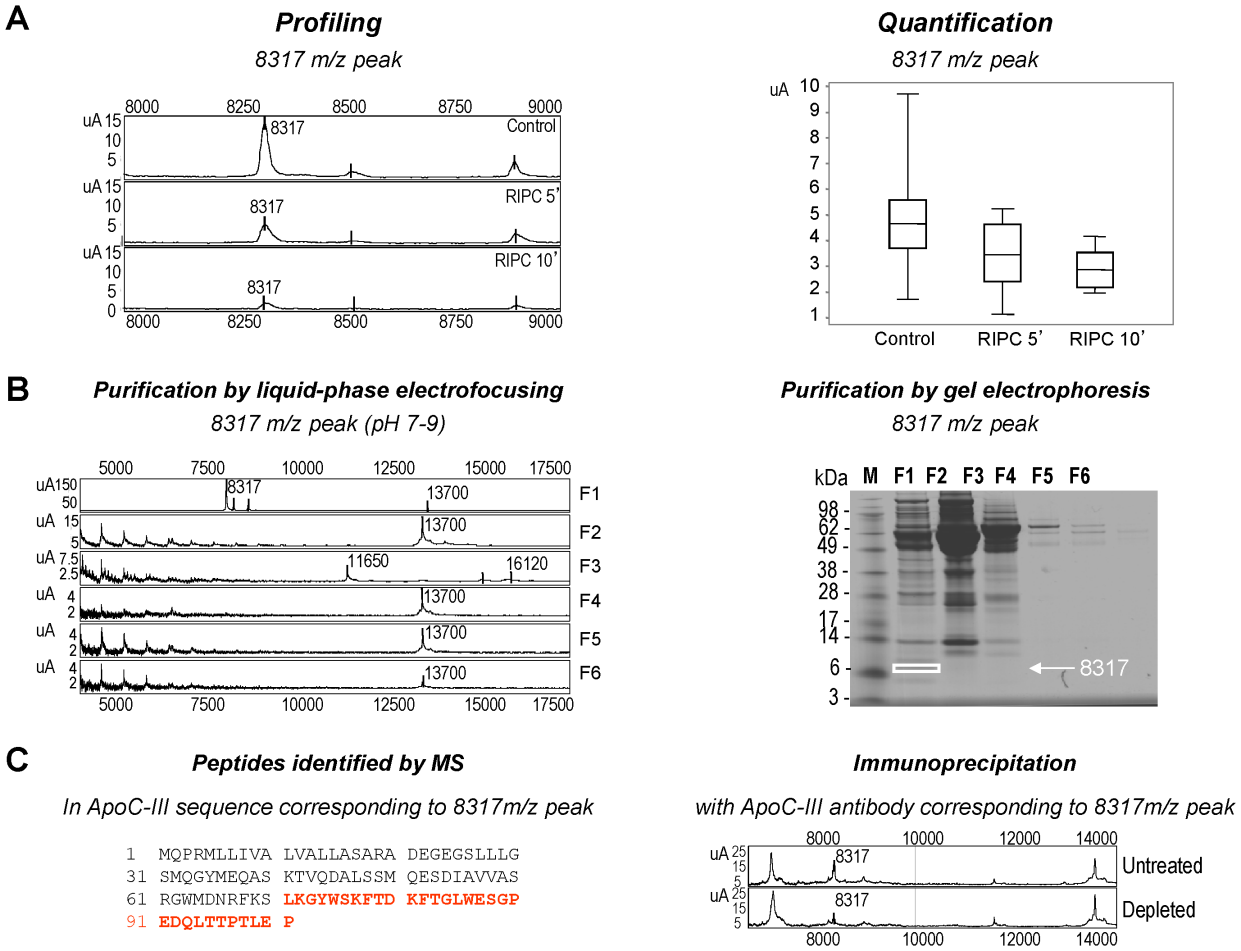
Profiling, purification and identification of the protein corresponding to the 8317 *m/z* peak. **A:** Representative SELDI-TOF-MS protein spectra of plasma sample from one Control, one RIPC 5′, and one RIPC 10′ rat. Results are presented as intensities of SELDI-TOF reading (arbitrary units). The 8317 *m/z* peak was found to be differentially expressed on the CM10 array as calculated by the Mann-Whitney test (left panel). Scattergram showing the significant differences in intensity of 8317 *m/z* peak in plasma samples derived from Control, RIPC 5′, and RIPC 10′ rats. The continuous line represents the mean, and dots represent each individual rat (n = 10 in each group) (right panel). Detailed *p-*value data for comparison between the three groups is indicated in Table 2. **B:** Purification of the protein corresponding to the 8317 *m/z* peak. SELDI-TOF-MS protein spectrum of fractions F1, F2, F3, F4, F5 and F6 from MicroRotofor® cell, by a pH gradient 7–9 (left panel). Each fraction (F1, F2, F3, F4, F5 and F6) was analyzed on NU-PAGE 10% coomassie blue stained-gel. The band corresponding to the 8317 *m/z* peak was framed (right panel). **C:** Identification of the 8317 *m/z* peak by mass spectrometry. Aminoacids indicated in red corresponds to the peptides identified in Apolipoprotein C-III (ApoC-III) (left panel). SELDI-TOF-MS protein spectra of crude (untreated) and immunodepleted plasma (20 µL) with 10 µg ApoC-III antibody (depleted) showed the decrease in 8317 *m/z* peak following immunodepletion, validating the identification (right panel).

**Figure 2A** presents a detailed representative spectrum of 8317 *m/z* peak for one rat from each group (left panel) and the mean intensity level in each group (right panel). Interestingly, we observed a decreased intensity for the peak at 10-min reperfusion compared to Control. Purification of the 8317 *m/z* peak by liquid-phase IEF (fraction F1) and gel electrophoresis (**Figure 2B**) combined with mass spectrometry successfully identified the 8317 *m/z* peak as being apolipoprotein C-III (ApoC-III) (**Figure 2C**). We verified the identification of 8317 peak using a specific polyclonal antibody. Immunodepletion significantly reduced the peak, thereby confirming the identification of the 8317 *m/z* peak as ApoC-III (**Figure 2C**).

Data concerning the others peaks are presented in supplemental section (**Figures S1–S7 in File S1**).

## Discussion

Our study’s major finding is that RIPC induced significant modulations in blood’s protein composition. We used the SELDI-TOF-MS method on ProteinChip Arrays, well known for its potential to identify circulating biomarkers of cardiovascular diseases [22], [25]. The CPLL was also employed with the goal of reducing high-abundance proteins and increasing the concentration of rare species. These low- and very low-abundance proteins, called deep proteomes, constitute the vast majority of human proteomes, and their analysis is an undeniable asset in the quest to identify the circulating factors of RIPC [26], [28]. The SELDI-TOF-MS analysis revealed a significant modification in plasma proteome following RIPC stimulus, with 296 peaks differentially expressed. Of these peaks, 100 were detected in native plasma, 115 in CPLL-treated plasma, and 81 in both forms of plasma. These results confirmed the relevance of plasma proteome analysis for the identification of circulating factors of RIPC. Finally, we found several proteins significantly modulated in response to RIPC.

These findings accord with numerous studies suggesting the key role of humoral mediators in RIPC signalling [18], [20]. Indeed, the mechanism for conveying cardioprotective information to the heart is still unknown, though many authors suggest that circulating factors could be released from the remote organ [16], [17], [19]. One proteomic study sought to identify the circulating factors of RIPC, but the results were inconclusive [24]. Having failed to successfully identify a cardioprotective protein, biochemical studies were then performed to characterize the chemical nature and molecular size of the circulating factors. The consensus is that the humoral factor is hydrophobic, and sized between 3.5 and 30 kDa [19], [24], [30], [31]. Over the last few years, proteomic and genomic techniques have been used to better define gene and protein expression modulations in response to RIPC stimulus [32]. Hepponstall *et al.* recently demonstrated that the transient I/R of an arm modifies plasma protein content in healthy volunteers. They identified 51 proteins significantly modulated after RIPC, strengthening the hypothesis of cardioprotective circulating RIPC factors [33]. In the same way, our study sought to identify plasma proteome modifications in rats following RIPC stimulus. We identified seven proteins that were significantly modulated after RIPC and involved in hemostasis, lipid transport, iron regulation, and the anti-inflammatory process (**Table 3**). Our group recently published the identification of apolipoprotein A-I (ApoA-I) as a potential RIPC-induced circulating factor [29]. ApoA-I, shown to be significantly increased in rat plasma following RIPC stimulus, was able to recapitulate the cardioprotection offered by RIPC when injected before myocardial I/R. Interestingly, some of the proteins identified in the present study are linked to this protein’s homeostasis. Notably, most of the proteins identified have a molecular size below 30 kDa, in accordance with the humoral RIPC factors’ hypothesized size.

**Table 3:** Main functions of regulated proteins after RIPC.

Protein name	Molecular mass	Modulation	Main function
	(Da)		
Haptoglobin alpha chain	9317	Up-regulated	Anti-oxidative and anti-inflammation
			ApoA-I protection
Apolipoprotein C-III	11117	Down-regulated	Lipid metabolism
			Pro-inflammation and atherogenesis
Transthyretin	15720	Up-regulated	Thyroid hormones, retinol and vitamin A transport
*(monomeric form)*			
Transthyretin	31440	Down-regulated	Anti-inflammation and ApoA-I binding in HDL
*(dimeric form)*			
Hemoglobin beta chain	15979	Down-regulated	Oxygen transport
Apolipoprotein A-IV	44456	Down-regulated	Lipid metabolism
			Anti-oxidative and anti-atherogenesis
Fibrinogen beta chain	50671	Down-regulated	Haemostasis, platelet aggregation
			Pro-inflammation

Haptoglobin (Hp) is a protein, synthesized in the liver, that scavenges hemoglobin (Hb) during hemolysis [34]. It is also known to exhibit strong anti-oxidant and anti-inflammatory properties, among them lymphocyte T helper-2 cytokine release and inhibition of cyclooxygenase [34], along with some tissular effects, like facilitating cell migration, angiogenesis, and tissue repair, and arterial restructuring by inhibition of gelatin degradation [35]. The involvement of Hp in I/R is not clearly described, but liver protection against I/R by pharmacological RIPC was related to a preservation of Hp expression, while I/R alone decreased Hp expression by 50% [36]. Furthermore, Hp was shown to bind to ApoA-I in order to protect its structure and function against hydroxyl radical-induced damages [37].

Transthyretin (TTR), or prealbumin, is a plasmatic protein synthesized in the liver and choroid plexus that is involved in the plasma and cerebrospinal fluid, carrying of thyroid hormones (T3, T4), retinol, and vitamin A [38]. In brain I/R models, TTR has neuroprotective effects, as TTR^−/−^ mice exhibit larger infarcts, increased edema formation, and delayed nerve regeneration [39]. TTR protective effects are mediated by the thyroid hormones carried, including reverse intracellular H^+^ accumulation after I/R, via stimulation of its efflux through a Na^+^/H^+^ exchanger. These events, occurring in the brain, are classically described in myocardial I/R injuries and could be extrapolated to cardioprotection of TTR through thyroid hormones. In addition to these cellular homeostatic effects, TTR seems to display anti-inflammatory properties via inhibition of monocyte and endothelial cells’ interleukine-1 production [40]. Furthermore, in humans, TTR levels are significantly lower in patients with acute myocardial infarction [38], suggesting increase in TTR levels may have a cardioprotective effect in RIPC. TTR is a 14 kDa protein present in different homomeric forms, such as dimeric (dTTR; 28 kDa) and monomeric (mTTR; 14 kDa) forms, in human plasma. Levels of mTTR have been shown to be significantly decreased in plasma from patients with familiar hypercholesterolemia in conjunction with episodes of cardiovascular disease, pointing to a protective effect of mTTR [38]. Interestingly, these two forms were significantly modulated in the different groups of rats in our study, with an up-regulation of mTTR and a down-regulation of dTTR in RIPC. Our data is consistent with a previous study of Suzuyama *et al.* [41]. The authors also used a SELDI-TOF-MS approach in order to identify changes in the rat cerebrospinal fluid proteome after transient focal cerebral ischemia, and demonstrated the monomeric form of TTR to be significantly increased during the acute phase of reperfusion. Hepponstall *et al.* also described TTR as over-expressed following RIPC in humans [33]. Interestingly, TTR is associated to ApoA-I in high-density lipoproteins (HDL) [42], and the only form carried by HDL is mTTR [38]. TTR, thus, has protective properties on its own through its carrying of thyroid hormones.

Fibrinogen β (Fgβ) is a hemostatic protein promoting platelet adhesion during I/R that was found to be down-regulated in the RIPC group. Platelet recruitment is induced by ICAM-1 expression on ischemic endothelial cells and leads to coronary reocclusion and an inflammatory response via ROS and cytokine release [43]. Its reduction could be related to protective effects of RIPC. Fgβ is transformed into fibrin β (Fβ) by thrombin, and then into the fibrin-derived peptide Bβ_15–42_ (Bβ_15–42_). Bβ_15–42_ corresponds to amino acids situated on the β-chain of Fβ, binding to VE-cadherin. In myocardial and kidney I/R, this peptide prevents interaction with leukocytes and their infiltration into the infarcted tissue [44]. Bβ_15–42_ has also been shown to reduce infarct size and production of IL-1β, TNF-α, and IL-6 [44]–[47]. These results evince the role of Fgβ in cardioprotection. The decrease in Fgβ in the RIPC group that our study observed suggests a degradation of Fβ in Bβ_15–42_ by RIPC. As the *m/z* detectable range limit in the SELDI-TOF-MS analysis was 3000 Da, we can speculate that the Bβ_15–42_ peptide was too small to be detected.

Apolipoprotein C-III (ApoC-III) is a small plasma protein of 8 kDa, synthezised in the liver, that presents mainly in very low density lipoproteins (VLDL) but also in low density lipoproteins (LDL) and HDL. Involved in triglyceride-rich lipoprotein clearance and catabolism, ApoC-III is associated with hypertriglyceridemia, type-I diabetes, and coronary heart diseases [48]–[50]. ApoC-III also induces inflammation, via ICAM-1 expression and monocyte adhesion to endothelial cells, leading to atherosclerosis [51].

Overall, most of the proteins identified in our study are in accordance with a protective role of RIPC (**Table 3**). Some proteins play a role in anti-inflammation, oxidative stress inhibition, hemostasis, and tissue repair, and others have cardioprotective properties documented in the literature. ApoA-I has recently been described as a potential circulating factor of RIPC [29]. Our study presents clear evidence that RIPC can induce rapid modulation in different plasma proteins. We have only studied the early response to RIPC (5- and 10-min limb reperfusion), which explains why the majority of the identified proteins were up-regulated. Nevertheless, these results are consistent with Hepponstall *et al.* [33] who described a strong tendency toward protein up-regulation following RIPC, followed by a down-regulation at 15-min and 24 h reperfusion.

The use of the SELDI-TOF-MS, for its ability to identify circulating biomarkers in plasma, combined with CPLL treatment, for studying deep-proteome, was successful. We have identified potential mediators of RIPC, having taken their size into consideration. Some of these proteins may be able to mimic RIPC cardioprotection *in vivo*, which could be a major step in the use of cardioprotection in clinical practice. These proteins’ capacity to mimic cardioprotection induced by RIPC must still be validated.

### Limitation Section

The proteins identified in our study were obtained with a single algorithm of 10 min limb ischemia-10 min reperfusion. Whether other algorithms of remote conditioning would induce the same modifications of plasmatic proteome needs to be confirmed. Our study was conducted in a rat model of limb ischemia. These results have to be confirmed in other experimental models, and other animal species. The cardioprotective effects of these 7 proteins also need to be confirmed in a MI model.

## Conclusion

Our data shows that RIPC by a short episode of limb I/R *in vivo* was highly related to proteomic modulation, with up- and down-regulation of plasma protein expression. We identified seven significantly modulated proteins as potential blood-borne RIPC factors. This data supports the involvement of circulating mediators in signal transduction from remote organs to the heart.

## Supporting Information

File S1 **Figure S1. Profiling of the proteins corresponding to the** ***m/z*** **peaks.** Representative SELDI-TOF-MS protein spectra of plasma sample from one Control, one RIPC 5′, and one RIPC 10′ rat for the 13720, 27340, 42463–42427, 42610 and 54700 *m/z* peaks. Results are presented as intensities of SELDI-TOF reading (arbitrary units). The 27340, 42427 and 42610 *m/z* peaks were found to be differentially expressed on the H50 array and the 13720, 42463 and 54700 *m/z* peaks on the CM10 array. The statistical significance was calculated by the Mann-Whitney test. **Figure S2. Quantification of the proteins corresponding to the m/z peaks.** Scattergrams showing the significant differences in intensity of each peaks in plasma samples derived from Control, RIPC 5′, and RIPC 10′ rats. The continuous line represents the mean, and dots represent each individual rat (n = 10 in each group). Detailed *p-*value data for comparison between the three groups is indicated in Table 2. **Figure S3. Purification by liquid-phase electrofocusing.** Protein corresponding to the *m/z* peaks indicated were purified using the MicroRotofor® cell. SELDI-TOF-MS protein spectra analysis of fractions from pH gradient 7–9 for 13720, 42463–42427 and 42610 m/z peaks, pH gradient 3–10 for 27340 m/z peak and 5–7 for 54700 m/z peak. **Figure S4. Purification by gel electrophoresis.** Each fraction obtained by liquid-phase electrofocusing was analyzed on NU-PAGE 10% coomassie blue stained-gel. The band corresponding to the peak of interest was framed. **Figure S5. Identification of the proteins corresponding to the** ***m/z*** **peaks.** Identification of the *m/z* peaks purifed by gel electrophoresis by mass spectrometry. Aminoacids indicated in red corresponds to the peptides identified in the protein sequence. **Figure S6. Identification of the proteins corresponding to the m/z peaks.** SELDI-TOF-MS protein spectra of crude (untreated) and immunodepleted plasmas with antibodies (depleted) showed the decrease in the corresponding *m/z* peak following immunodepletion, validating the identification. **Figure S7. Profiling and verification of identified proteins corresponding to the 9420 and 15870–15980** ***m/z*** **peaks. A:** Representative SELDI-TOF-MS protein spectra of plasma sample from one Control, one RIPC 5′, and one RIPC 10′ rat. Results are presented as intensities of SELDI-TOF reading (arbitrary units). **B:** The 9420 and 15870–15980**** *m/z* peak were respectively found to be differentially expressed on the H50 and CM10 arrays as calculated by the Mann-Whitney test. Scattergram showing the significant differences in intensity of 9420 (left panel), 15870 (middle panel) and 15980 (right panel) *m/z* peaks in plasma samples derived from Control, RIPC 5′, and RIPC 10′ rats. The continuous line represents the mean, and dots represent each individual rat (n = 10 in each group). Detailed *p-*value data for comparison between the three groups is indicated in Table 2. **C:** SELDI-TOF-MS protein spectra of crude (untreated) and immunodepleted plasma (10 µL) with 10 µg of haptoglobin or 5 µg of hemoglobin antibody (depleted) showed the decrease in 9420 and 15870–15980 *m/z* peak following immunodepletion, validating the identifications.
(DOCX)

## References

[1] Murry CE, Jennings RB, Reimer KA (1986) Preconditioning with ischemia: a delay of lethal cell injury in ischemic myocardium. Circulation 74: 1124–1136. 3769170 10.1161/01.cir.74.5.1124

[2] Jenkins DP, Pugsley WB, Alkhulaifi AM, Kemp M, Hooper J, et al (1997) Ischaemic preconditioning reduces troponin T release in patients undergoing coronary artery bypass surgery. Heart 77: 314–318. 9155608 10.1136/hrt.77.4.314 PMC484723

[3] Walsh SR, Tang TY, Kullar P, Jenkins DP, Dutka DP, et al (2008) Ischaemic preconditioning during cardiac surgery: systematic review and meta-analysis of perioperative outcomes in randomised clinical trials. Eur J Cardiothorac Surg 34: 985–994. 18783958 10.1016/j.ejcts.2008.07.062

[4] Przyklenk K, Bauer B, Ovize M, Kloner RA, Whittaker P (1993) Regional ischemic ‘preconditioning’ protects remote virgin myocardium from subsequent sustained coronary occlusion. Circulation 87: 893–899. 7680290 10.1161/01.cir.87.3.893

[5] Schoemaker RG, van Heijningen CL (2000) Bradykinin mediates cardiac preconditioning at a distance. Am J Physiol Heart Circ Physiol 278: H1571–1576. 10775135 10.1152/ajpheart.2000.278.5.H1571

[6] Pell TJ, Baxter GF, Yellon DM, Drew GM (1998) Renal ischemia preconditions myocardium: role of adenosine receptors and ATP-sensitive potassium channels. Am J Physiol 275: H1542–1547. 9815059 10.1152/ajpheart.1998.275.5.H1542

[7] Birnbaum Y, Hale SL, Kloner RA (1997) Ischemic preconditioning at a distance: reduction of myocardial infarct size by partial reduction of blood supply combined with rapid stimulation of the gastrocnemius muscle in the rabbit. Circulation 96: 1641–1646. 9315559 10.1161/01.cir.96.5.1641

[8] Kharbanda RK, Mortensen UM, White PA, Kristiansen SB, Schmidt MR, et al (2002) Transient limb ischemia induces remote ischemic preconditioning in vivo. Circulation 106: 2881–2883. 12460865 10.1161/01.cir.0000043806.51912.9b

[9] Brevoord D, Kranke P, Kuijpers M, Weber N, Hollmann M, et al (2012) Remote ischemic conditioning to protect against ischemia-reperfusion injury: a systematic review and meta-analysis. PLoS One 7: e42179. 22860077 10.1371/journal.pone.0042179 PMC3409156

[10] Cheung MM, Kharbanda RK, Konstantinov IE, Shimizu M, Frndova H, et al (2006) Randomized controlled trial of the effects of remote ischemic preconditioning on children undergoing cardiac surgery: first clinical application in humans. J Am Coll Cardiol 47: 2277–2282. 16750696 10.1016/j.jacc.2006.01.066

[11] Hausenloy DJ, Mwamure PK, Venugopal V, Harris J, Barnard M, et al (2007) Effect of remote ischaemic preconditioning on myocardial injury in patients undergoing coronary artery bypass graft surgery: a randomised controlled trial. Lancet 370: 575–579. 17707752 10.1016/S0140-6736(07)61296-3

[12] Thielmann M, Kottenberg E, Boengler K, Raffelsieper C, Neuhaeuser M, et al (2010) Remote ischemic preconditioning reduces myocardial injury after coronary artery bypass surgery with crystalloid cardioplegic arrest. Basic Res Cardiol 105: 657–664. 20495811 10.1007/s00395-010-0104-5

[13] Ali ZA, Callaghan CJ, Lim E, Ali AA, Nouraei SA, et al (2007) Remote ischemic preconditioning reduces myocardial and renal injury after elective abdominal aortic aneurysm repair: a randomized controlled trial. Circulation 116: I98–105. 17846333 10.1161/circulationaha.106.679167

[14] Hoole SP, Heck PM, Sharples L, Khan SN, Duehmke R, et al (2009) Cardiac Remote Ischemic Preconditioning in Coronary Stenting (CRISP Stent) Study: a prospective, randomized control trial. Circulation 119: 820–827. 19188504 10.1161/CIRCULATIONAHA.108.809723

[15] Botker HE, Kharbanda R, Schmidt MR, Bottcher M, Kaltoft AK, et al (2010) Remote ischaemic conditioning before hospital admission, as a complement to angioplasty, and effect on myocardial salvage in patients with acute myocardial infarction: a randomised trial. Lancet 375: 727–734. 20189026 10.1016/S0140-6736(09)62001-8

[16] Dickson EW, Reinhardt CP, Renzi FP, Becker RC, Porcaro WA, et al (1999) Ischemic preconditioning may be transferable via whole blood transfusion: preliminary evidence. J Thromb Thrombolysis 8: 123–129. 10436142 10.1023/a:1008911101951

[17] Dickson EW, Lorbar M, Porcaro WA, Fenton RA, Reinhardt CP, et al (1999) Rabbit heart can be “preconditioned” via transfer of coronary effluent. Am J Physiol 277: H2451–2457. 10600868 10.1152/ajpheart.1999.277.6.H2451

[18] Konstantinov IE, Li J, Cheung MM, Shimizu M, Stokoe J, et al (2005) Remote ischemic preconditioning of the recipient reduces myocardial ischemia-reperfusion injury of the denervated donor heart via a Katp channel-dependent mechanism. Transplantation 79: 1691–1695. 15973170 10.1097/01.tp.0000159137.76400.5d

[19] Shimizu M, Tropak M, Diaz RJ, Suto F, Surendra H, et al (2009) Transient limb ischaemia remotely preconditions through a humoral mechanism acting directly on the myocardium: evidence suggesting cross-species protection. Clin Sci (Lond) 117: 191–200. 19175358 10.1042/CS20080523

[20] Breivik L, Helgeland E, Aarnes EK, Mrdalj J, Jonassen AK (2011) Remote postconditioning by humoral factors in effluent from ischemic preconditioned rat hearts is mediated via PI3K/Akt-dependent cell-survival signaling at reperfusion. Basic Res Cardiol 106: 135–145. 21103992 10.1007/s00395-010-0133-0 PMC3012213

[21] Dubois E, Richard V, Mulder P, Lamblin N, Drobecq H, et al (2011) Decreased serine207 phosphorylation of troponin T as a biomarker for left ventricular remodelling after myocardial infarction. Eur Heart J 32: 115–123. 20418543 10.1093/eurheartj/ehq108

[22] Pinet F, Beseme O, Cieniewski-Bernard C, Drobecq H, Jourdain S, et al (2008) Predicting left ventricular remodeling after a first myocardial infarction by plasma proteome analysis. Proteomics 8: 1798–1808. 18384103 10.1002/pmic.200700781

[23] Acosta-Martin AE, Panchaud A, Chwastyniak M, Dupont A, Juthier F, et al (2011) Quantitative mass spectrometry analysis using PAcIFIC for the identification of plasma diagnostic biomarkers for abdominal aortic aneurysm. PLoS One 6: e28698. 22163325 10.1371/journal.pone.0028698 PMC3233585

[24] Lang SC, Elsasser A, Scheler C, Vetter S, Tiefenbacher CP, et al (2006) Myocardial preconditioning and remote renal preconditioning–identifying a protective factor using proteomic methods? Basic Res Cardiol 101: 149–158. 16283592 10.1007/s00395-005-0565-0

[25] Fertin M, Beseme O, Duban S, Amouyel P, Bauters C, et al (2010) Deep plasma proteomic analysis of patients with left ventricular remodeling after a first myocardial infarction. Proteomics Clin Appl 4: 654–673. 21137084 10.1002/prca.200900178

[26] Fertin M, Burdese J, Beseme O, Amouyel P, Bauters C, et al (2011) Strategy for purification and mass spectrometry identification of SELDI peaks corresponding to low-abundance plasma and serum proteins. J Proteomics 74: 420–430. 21184852 10.1016/j.jprot.2010.12.005

[27] Neuhoff V, Arold N, Taube D, Ehrhardt W (1988) Improved staining of proteins in polyacrylamide gels including isoelectric focusing gels with clear background at nanogram sensitivity using Coomassie Brilliant Blue G-250 and R-250. Electrophoresis 9: 255–262. 2466658 10.1002/elps.1150090603

[28] Beseme O, Fertin M, Drobecq H, Amouyel P, Pinet F (2010) Combinatorial peptide ligand library plasma treatment: Advantages for accessing low-abundance proteins. Electrophoresis 31: 2697–2704. 20665525 10.1002/elps.201000188

[29] Hibert P, Prunier-Mirebeau D, Beseme O, Chwastyniak M, Tamareille S, et al (2013) Apolipoprotein A-I Is a Potential Mediator of Remote Ischemic Preconditioning. PLoS One 8: e77211. 24155931 10.1371/journal.pone.0077211 PMC3796499

[30] Dickson EW, Blehar DJ, Carraway RE, Heard SO, Steinberg G, et al (2001) Naloxone blocks transferred preconditioning in isolated rabbit hearts. J Mol Cell Cardiol 33: 1751–1756. 11549353 10.1006/jmcc.2001.1436

[31] Serejo FC, Rodrigues LF Jr, da Silva Tavares KC, de Carvalho AC, Nascimento JH (2007) Cardioprotective properties of humoral factors released from rat hearts subject to ischemic preconditioning. J Cardiovasc Pharmacol 49: 214–220. 17438406 10.1097/FJC.0b013e3180325ad9

[32] Konstantinov IE, Arab S, Kharbanda RK, Li J, Cheung MM, et al (2004) The remote ischemic preconditioning stimulus modifies inflammatory gene expression in humans. Physiol Genomics 19: 143–150. 15304621 10.1152/physiolgenomics.00046.2004

[33] Hepponstall M, Ignjatovic V, Binos S, Monagle P, Jones B, et al (2012) Remote Ischemic Preconditioning (RIPC) Modifies Plasma Proteome in Humans. PLoS One 7: e48284. 23139772 10.1371/journal.pone.0048284 PMC3489679

[34] Quaye IK (2008) Haptoglobin, inflammation and disease. Trans R Soc Trop Med Hyg 102: 735–742. 18486167 10.1016/j.trstmh.2008.04.010

[35] de Kleijn DP, Smeets MB, Kemmeren PP, Lim SK, Van Middelaar BJ, et al (2002) Acute-phase protein haptoglobin is a cell migration factor involved in arterial restructuring. FASEB J 16: 1123–1125. 12039846 10.1096/fj.02-0019fje

[36] Fernandez V, Castillo I, Tapia G, Romanque P, Uribe-Echevarria S, et al (2007) Thyroid hormone preconditioning: protection against ischemia-reperfusion liver injury in the rat. Hepatology 45: 170–177. 17187421 10.1002/hep.21476

[37] Salvatore A, Cigliano L, Bucci EM, Corpillo D, Velasco S, et al (2007) Haptoglobin binding to apolipoprotein A-I prevents damage from hydroxyl radicals on its stimulatory activity of the enzyme lecithin-cholesterol acyl-transferase. Biochemistry 46: 11158–11168. 17824618 10.1021/bi7006349

[38] Cubedo J, Padro T, Alonso R, Cinca J, Mata P, et al (2012) Differential proteomic distribution of TTR (pre-albumin) forms in serum and HDL of patients with high cardiovascular risk. Atherosclerosis 222: 263–269. 22420894 10.1016/j.atherosclerosis.2012.02.024

[39] Santos SD, Lambertsen KL, Clausen BH, Akinc A, Alvarez R, et al (2010) CSF transthyretin neuroprotection in a mouse model of brain ischemia. J Neurochem 115: 1434–1444. 21044072 10.1111/j.1471-4159.2010.07047.x

[40] Borish L, King MS, Mascali JJ, Johnson S, Coll B, et al (1992) Transthyretin is an inhibitor of monocyte and endothelial cell interleukin-1 production. Inflammation 16: 471–484. 1385328 10.1007/BF00918973

[41] Suzuyama K, Shiraishi T, Oishi T, Ueda S, Okamoto H, et al (2004) Combined proteomic approach with SELDI-TOF-MS and peptide mass fingerprinting identified the rapid increase of monomeric transthyretin in rat cerebrospinal fluid after transient focal cerebral ischemia. Brain Res Mol Brain Res 129: 44–53. 15469881 10.1016/j.molbrainres.2004.06.021

[42] Sousa MM, Berglund L, Saraiva MJ (2000) Transthyretin in high density lipoproteins: association with apolipoprotein A-I. J Lipid Res 41: 58–65. 10627502

[43] Massberg S, Enders G, Matos FC, Tomic LI, Leiderer R, et al (1999) Fibrinogen deposition at the postischemic vessel wall promotes platelet adhesion during ischemia-reperfusion in vivo. Blood 94: 3829–3838. 10572098

[44] Petzelbauer P, Zacharowski PA, Miyazaki Y, Friedl P, Wickenhauser G, et al (2005) The fibrin-derived peptide Bbeta15–42 protects the myocardium against ischemia-reperfusion injury. Nat Med 11: 298–304. 15723073 10.1038/nm1198

[45] Krishnamoorthy A, Ajay AK, Hoffmann D, Kim TM, Ramirez V, et al (2011) Fibrinogen beta-derived Bbeta(15–42) peptide protects against kidney ischemia/reperfusion injury. Blood 118: 1934–1942. 21685370 10.1182/blood-2011-02-338061 PMC3158721

[46] Roesner JP, Petzelbauer P, Koch A, Mersmann J, Zacharowski PA, et al (2007) The fibrin-derived peptide Bbeta15–42 is cardioprotective in a pig model of myocardial ischemia-reperfusion injury. Crit Care Med 35: 1730–1735. 17522584 10.1097/01.CCM.0000269035.30231.76

[47] Zacharowski K, Zacharowski PA, Friedl P, Mastan P, Koch A, et al (2007) The effects of the fibrin-derived peptide Bbeta(15–42) in acute and chronic rodent models of myocardial ischemia-reperfusion. Shock 27: 631–637. 17505302 10.1097/SHK.0b013e31802fa038

[48] Gerber Y, Goldbourt U, Segev S, Harats D (2003) Indices related to apo CII and CIII serum concentrations and coronary heart disease: a case-control study. Prev Med 37: 18–22. 12799125 10.1016/s0091-7435(03)00051-3

[49] Ooi EM, Barrett PH, Chan DC, Watts GF (2008) Apolipoprotein C-III: understanding an emerging cardiovascular risk factor. Clin Sci (Lond) 114: 611–624. 18399797 10.1042/CS20070308

[50] Yao Z, Wang Y (2012) Apolipoprotein C-III and hepatic triglyceride-rich lipoprotein production. Curr Opin Lipidol 23: 206–212. 22510806 10.1097/MOL.0b013e328352dc70

[51] Kawakami A, Yoshida M (2009) Apolipoprotein CIII links dyslipidemia with atherosclerosis. J Atheroscler Thromb 16: 6–11. 19262004 10.5551/jat.e607

