# Supplementary material for: Modifications in Rat Plasma Proteome after Remote Ischemic Preconditioning (RIPC) Stimulus: Identification by a SELDI-TOF-MS Approach
Source: PLoS One. 2014 Jan 13;9(1):e85669. doi: 10.1371/journal.pone.0085669 (PMC3890329; doi:10.1371/journal.pone.0085669)

**SUPPORTING INFORMATION**

## **Protein identification of the peaks differentially expressed between the three groups of rats**

**Figure S1** presents a detailed representative spectrum of all the 13720, 27340, 42463, 42427-42610 and 54700 *m/z* peaks for one rat from each group and **Figure S2** the mean intensity level in each group. Liquid-phase IEF (**Figure S3**) and gel electrophoresis (**Figure S4**) successfully purified the *m/z* peaks and combination with mass spectrometry (**Figure S5**) identified the *m/z* peak as being transthyretin (13720 and 27340 *m/z*), apolipoprotein A-IV (42463-42427 and 42610 *m/z*) and fibrinogen beta chain (54700 *m/z*). We verified the identification using specific antibodies. Immunodepletion significantly reduced the peaks, thereby confirming the identifications (**Figure S6**).

We also checked from a recent publication in which a systematic identification of SELDI peaks was performed by MALDI-TOF {Albrethsen, 2011 #2888} whether some of the peaks selected to be differentially expressed have already been identified. And in that case confirmation of the identity of protein needs to be tested by immunoprecipitation with a specific antibody against the protein.

The 9420 *m/z* peak has been identified to be apolipoprotein C-III (ApoC-III) {Albrethsen, 2011 #2888}. This peak is up-regulated between RIPC rats and controls but the difference is only statistically different between CRTL and RIPC 10’ groups (**Figure S7A-B, Table 2**). We used a specific antibody against ApoC-III to confirm the identity, but immunodepletion of plasma sample did not deplete the peak, excluding ApoC-III to be the corresponding protein (data not shown).

By immunodepletion performed with an antibody against haptoglobin, we confirmed the identification of 9420 *m/z* peak to be haptoglobin alpha chain (**Figure S7 C (left panel)**).

The 15870-15980 *m/z* peaks have been previously identified by us in another study to be hemoglobin {Pinet, 2008 #717}. These peaks are up-regulated between controls and RIPC 5’ rats and down regulated in RIPC 10’ rats compared to the other groups but the difference is only statistically different between RIPC 5’ and RIPC 10’ groups (**Figure S7 A-B, Table 2**). By immunodepletion performed with a specific antibody against hemoglobin, we confirmed the identification of 15870-15980 *m/z* peaks to be hemoglobin beta chain (**Figure S7C, right panel**).


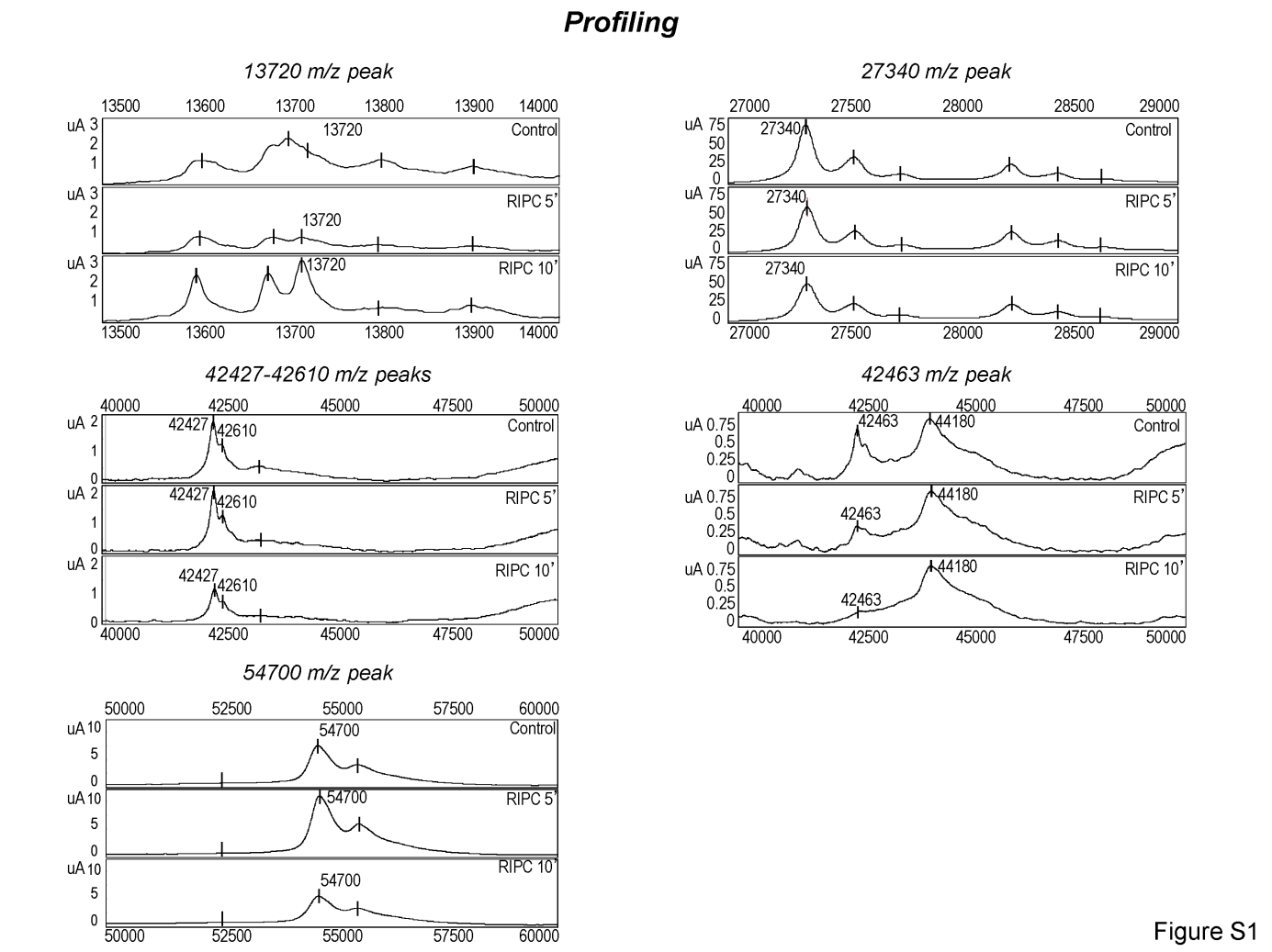


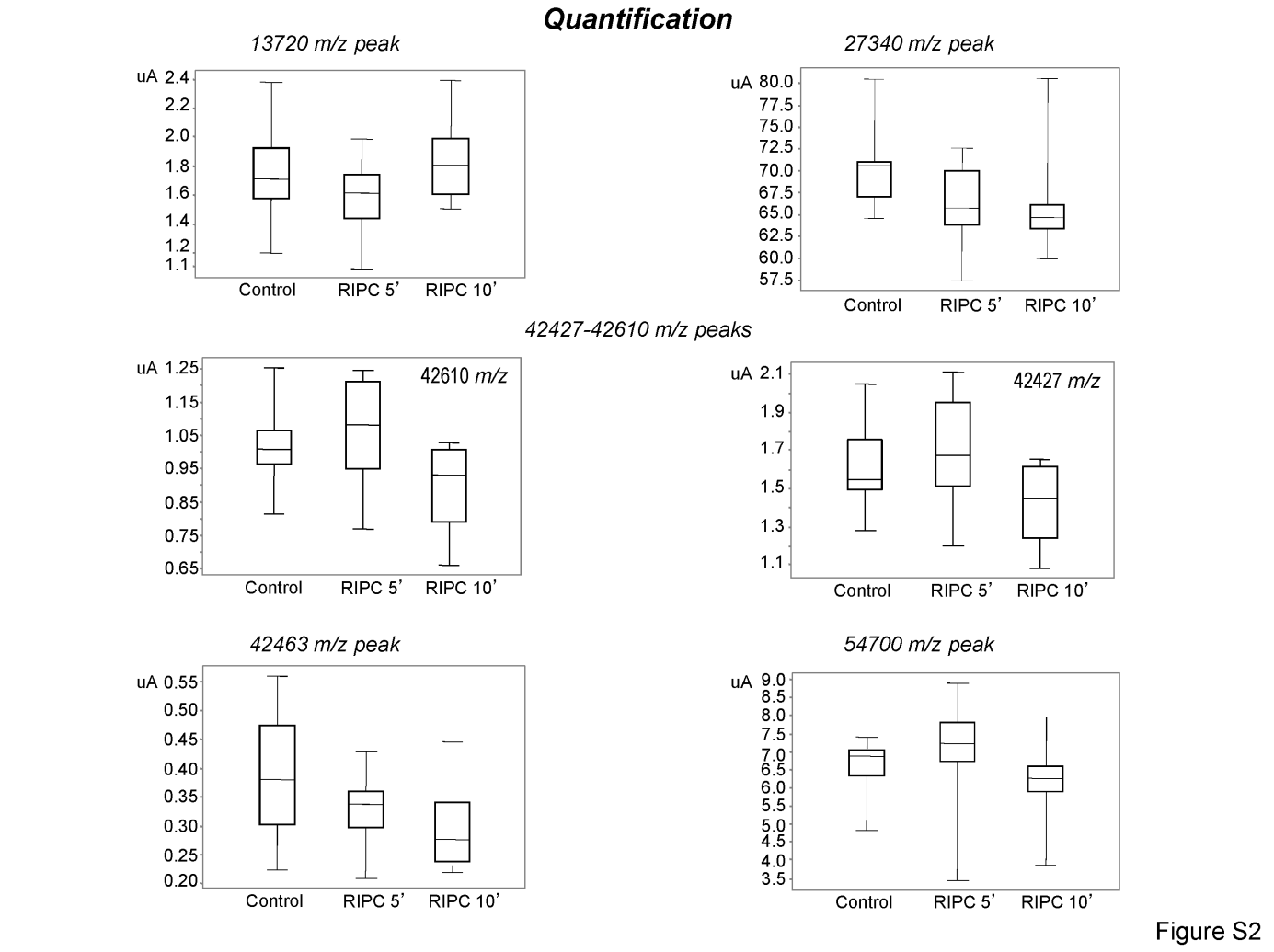


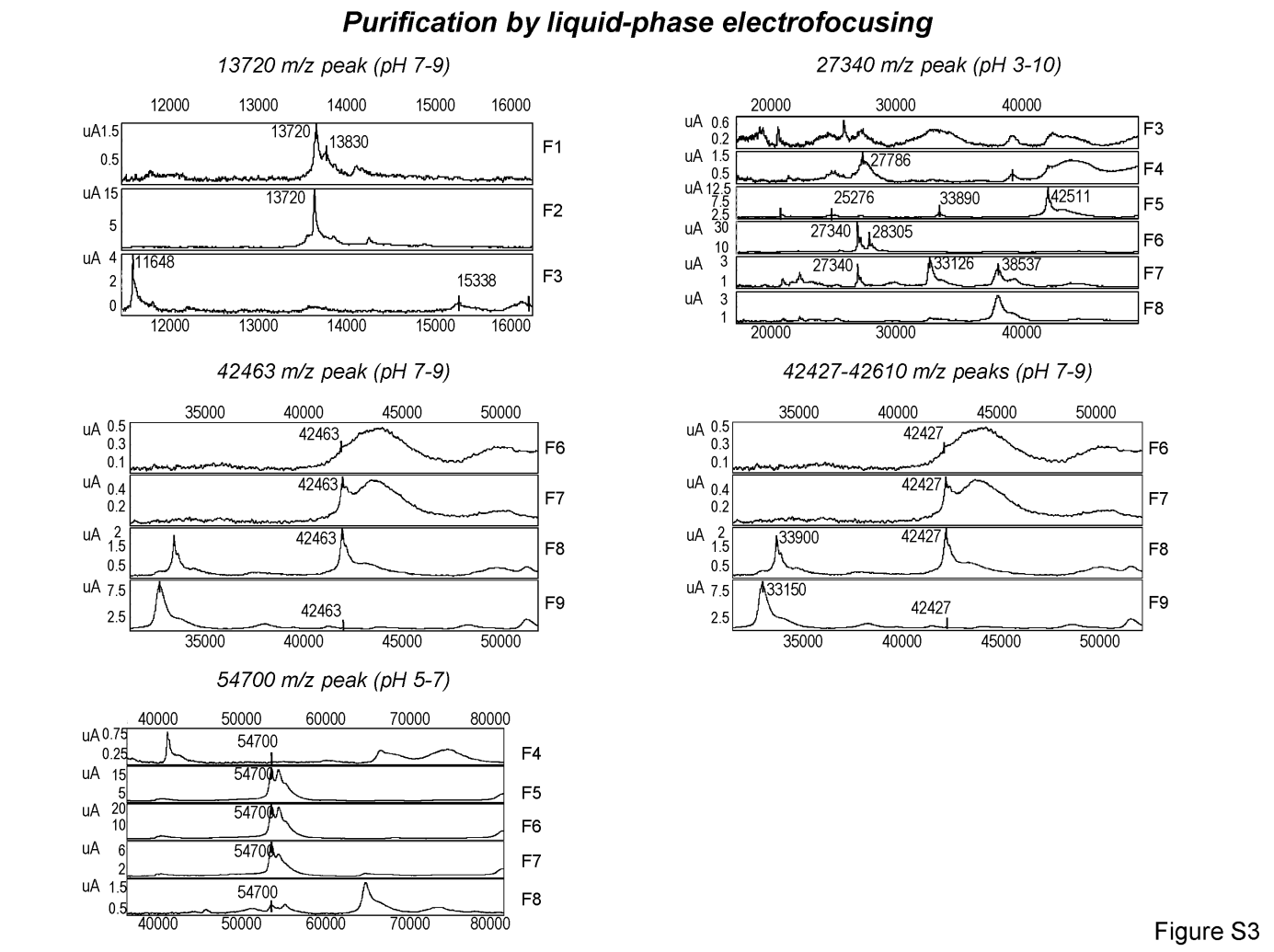


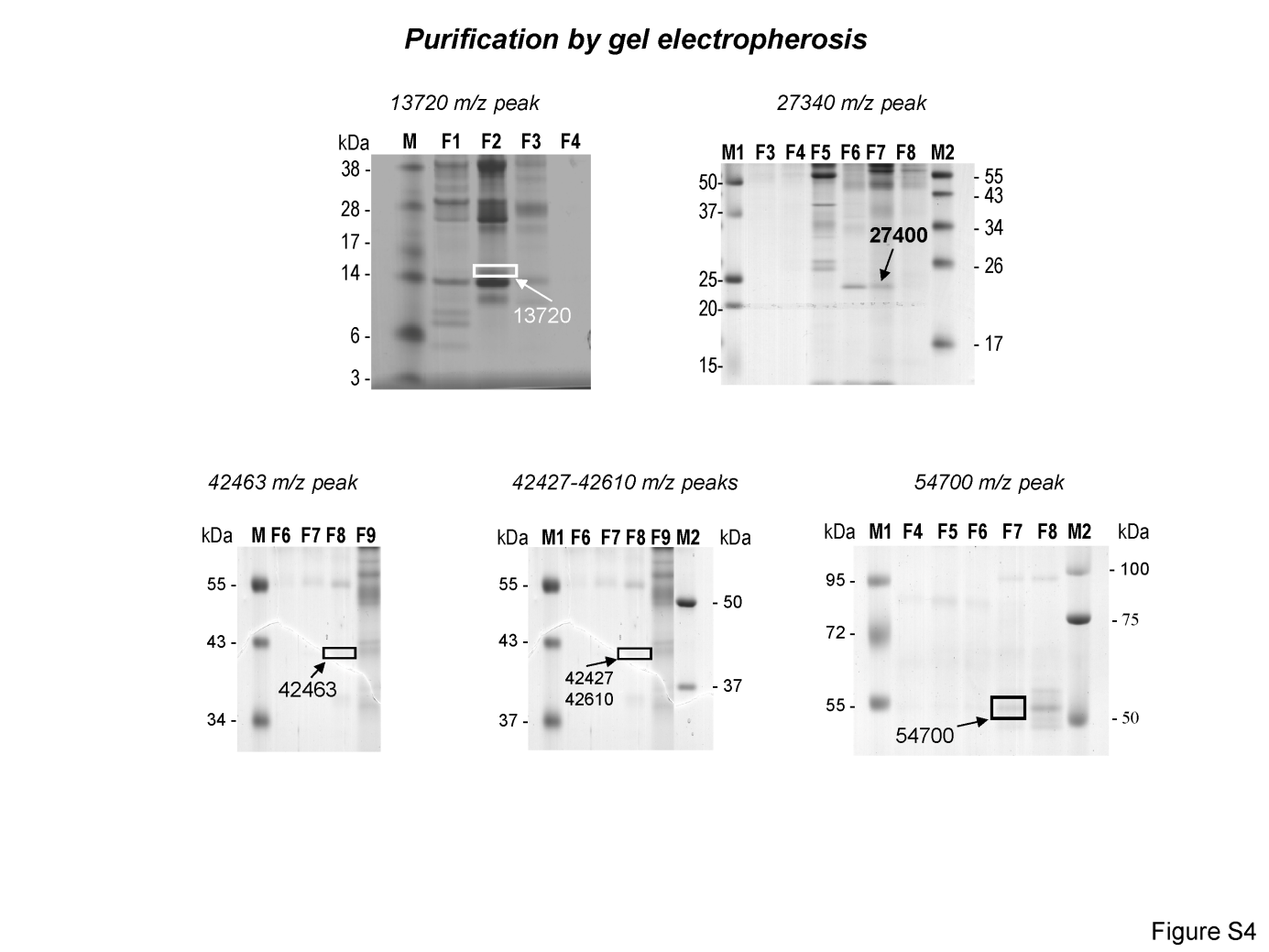


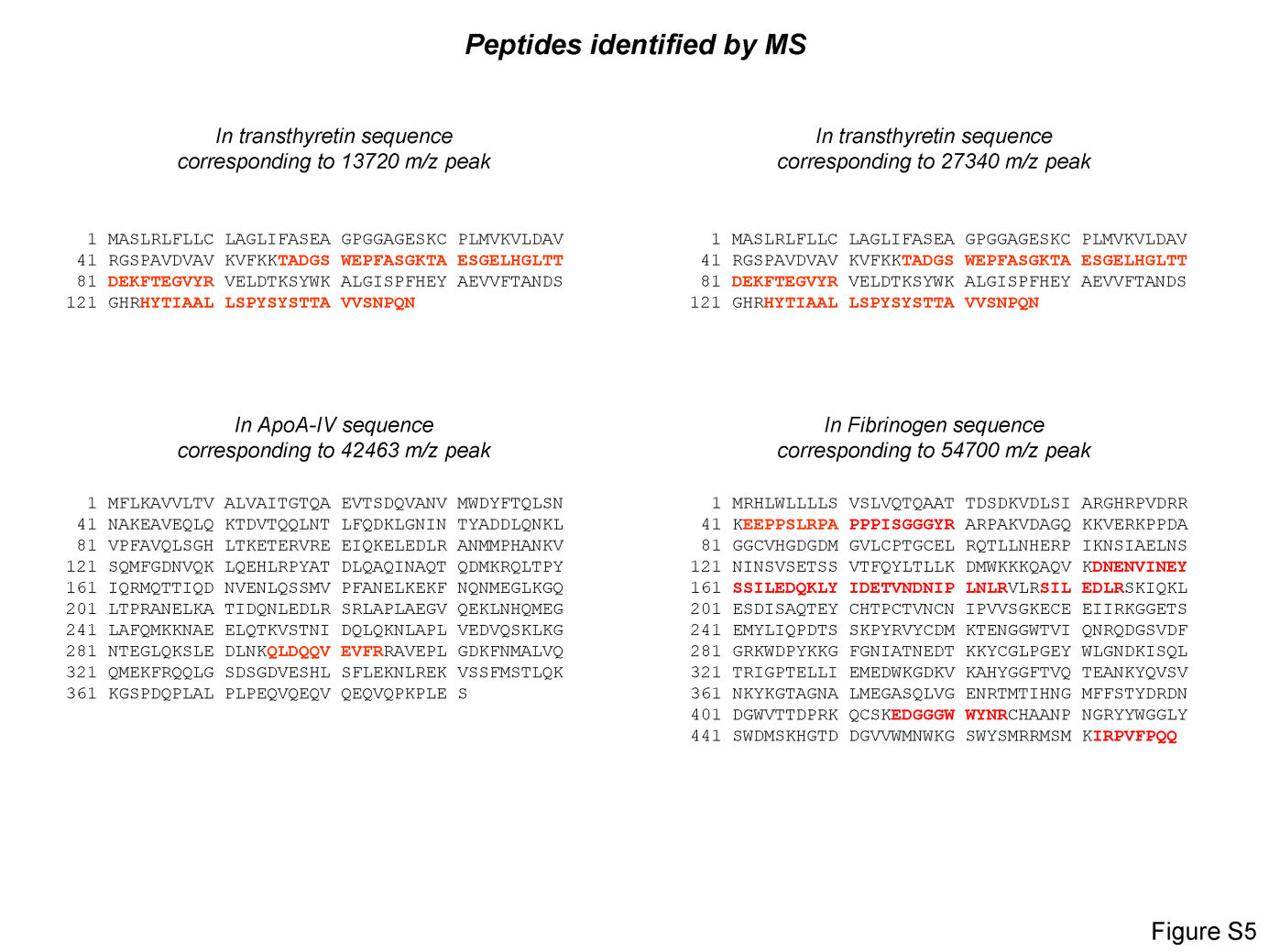


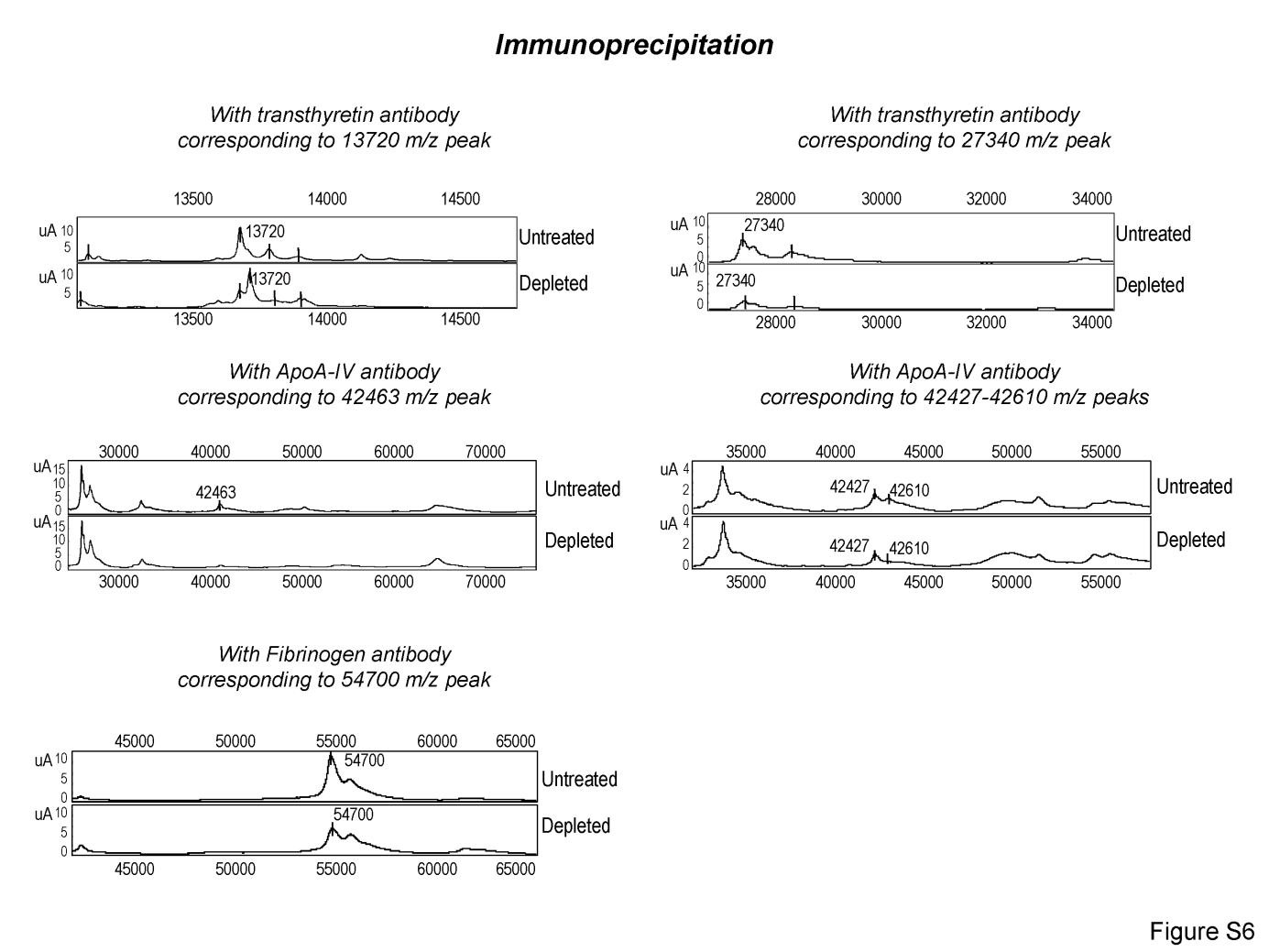


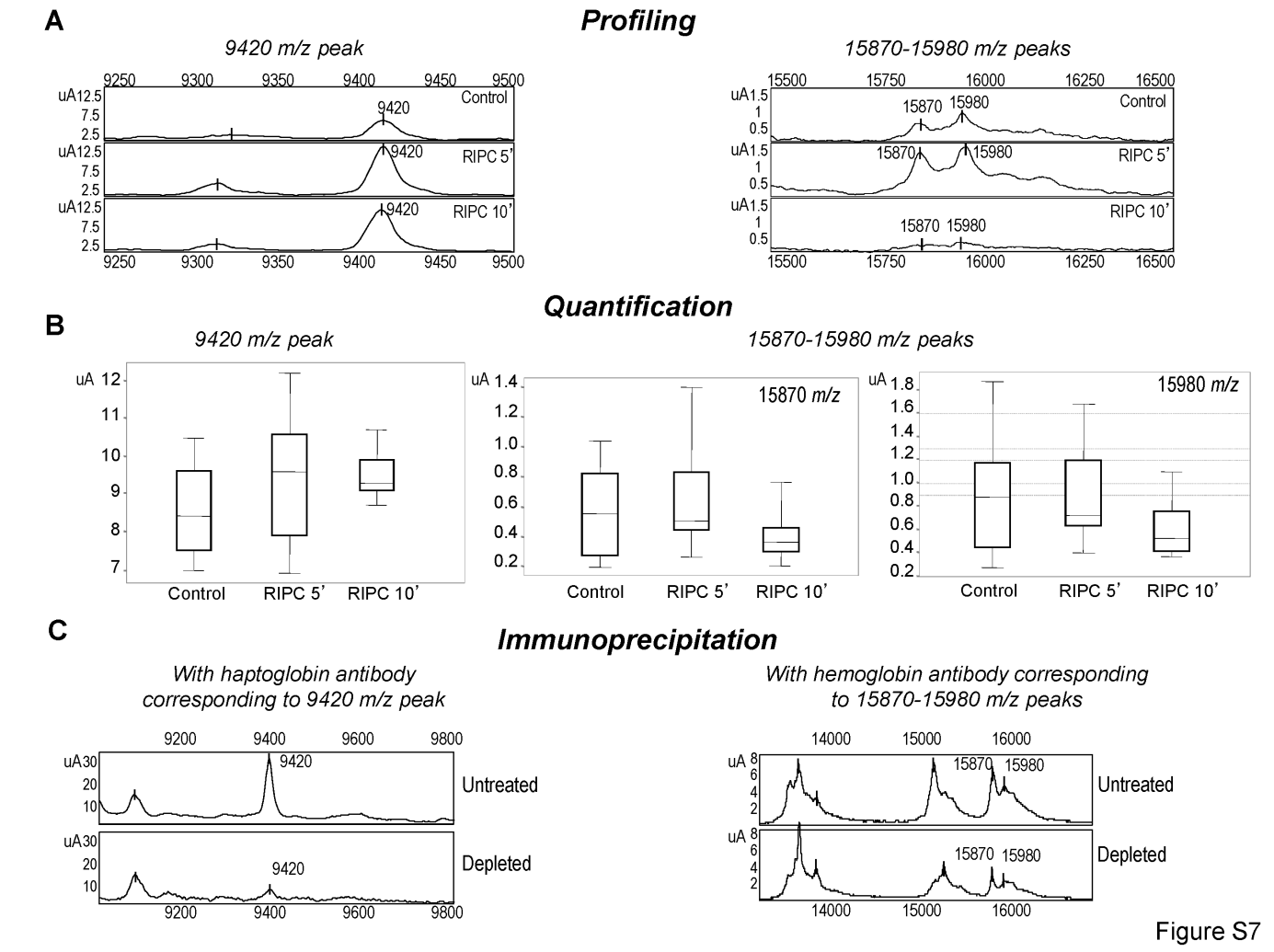

Supplement: File S1 — Figure S1. Profiling of the proteins corresponding to the m/z peaks. Representative SELDI-TOF-MS protein spectra of plasma sample from one Control, one RIPC 5′, and one RIPC 10′ rat for the 13720, 27340, 42463–42427, 42610 and 54700 m/z peaks. Results are presented as intensities of SELDI-TOF reading (arbitrary units). The 27340, 42427 and 42610 m/z peaks were found to be differentially expressed on the H50 array and the 13720, 42463 and 54700 m/z peaks on the CM10 array. The statistical significance was calculated by the Mann-Whitney test. Figure S2. Quantification of the proteins corresponding to the m/z peaks. Scattergrams showing the significant differences in intensity of each peaks in plasma samples derived from Control, RIPC 5′, and RIPC 10′ rats. The continuous line represents the mean, and dots represent each individual rat (n = 10 in each group). Detailed p-value data for comparison between the three groups is indicated in Table 2. Figure S3. Purification by liquid-phase electrofocusing. Protein corresponding to the m/z peaks indicated were purified using the MicroRotofor® cell. SELDI-TOF-MS protein spectra analysis of fractions from pH gradient 7–9 for 13720, 42463–42427 and 42610 m/z peaks, pH gradient 3–10 for 27340 m/z peak and 5–7 for 54700 m/z peak. Figure S4. Purification by gel electrophoresis. Each fraction obtained by liquid-phase electrofocusing was analyzed on NU-PAGE 10% coomassie blue stained-gel. The band corresponding to the peak of interest was framed. Figure S5. Identification of the proteins corresponding to the m/z peaks. Identification of the m/z peaks purifed by gel electrophoresis by mass spectrometry. Aminoacids indicated in red corresponds to the peptides identified in the protein sequence. Figure S6. Identification of the proteins corresponding to the m/z peaks. SELDI-TOF-MS protein spectra of crude (untreated) and immunodepleted plasmas with antibodies (depleted) showed the decrease in the corresponding m/z peak following immunod [file pone.0085669.s001.docx]
